# Anticancer Imidazoacridinone C-1311 is Effective in Androgen-Dependent and Androgen-Independent Prostate Cancer Cells

**DOI:** 10.3390/biomedicines8090292

**Published:** 2020-08-19

**Authors:** Magdalena Niemira, Barbara Borowa-Mazgaj, Samuel B. Bader, Adrianna Moszyńska, Marcin Ratajewski, Kaja Karaś, Mirosław Kwaśniewski, Adam Krętowski, Zofia Mazerska, Ester M. Hammond, Anna Skwarska

**Affiliations:** 1 Clinical Research Centre, Medical University of Bialystok, 15-276 Bialystok, Poland; adamkretowski@wp.pl; 2 Division of Biochemical Toxicology, National Center for Toxicological Research, Jefferson, AR 72079, USA; barborowm@gmail.com; 3 Department of Oncology, University of Oxford, Oxford OX3 7DQ, UK; samuel.bader@oncology.ox.ac.uk (S.B.B.); ester.hammond@oncology.ox.ac.uk (E.M.H.); 4 Department of Biology and Pharmaceutical Botany, Medical University of Gdansk, 80-416 Gdansk, Poland; adrianna.moszynska@gumed.edu.pl; 5 Laboratory of Epigenetics, Institute of Medical Biology PAS, 93-232 Lodz, Poland; mratajewski@cbm.pan.pl (M.R.); kaja.karas@gmail.com (K.K.); 6 Centre for Bioinformatics and Data Analysis, Medical University of Bialystok, 15-276 Bialystok, Poland; miroslaw.kwasniewski@umb.edu.pl; 7 Department of Endocrinology, Diabetology and Internal Medicine, Medical University of Bialystok, 15-276 Bialystok, Poland; 8 Department of Pharmaceutical Technology and Biochemistry, Gdansk University of Technology, 80-233 Gdansk, Poland; zofia.mazerska@pg.edu.pl

**Keywords:** androgen receptor, prostate cancer, C-1311/Symadex™, transcriptomic profiling, next-generation sequencing

## Abstract

The androgen receptor (AR) plays a critical role in prostate cancer (PCa) development and metastasis. Thus, blocking AR activity and its downstream signaling constitutes a major strategy for PCa treatment. Here, we report on the potent anti-PCa activity of a small-molecule imidazoacridinone, C-1311. In AR-positive PCa cells, C-1311 was found to inhibit the transcriptional activity of AR, uncovering a novel mechanism that may be relevant for its anticancer effect. Mechanistically, C-1311 decreased the AR binding to the prostate-specific antigen (*PSA*) promoter, reduced the PSA protein level, and, as shown by transcriptome sequencing, downregulated numerous AR target genes. Importantly, AR-negative PCa cells were also sensitive to C-1311, suggesting a promising efficacy in the androgen-independent PCa sub-type. Irrespective of AR status, C-1311 induced DNA damage, arrested cell cycle progression, and induced apoptosis. RNA sequencing indicated significant differences in the transcriptional response to C-1311 between the PCa cells. Gene ontology analysis showed that in AR-dependent PCa cells, C-1311 mainly affected the DNA damage response pathways. In contrast, in AR-independent PCa cells, C-1311 targeted the cellular metabolism and inhibited the genes regulating glycolysis and gluconeogenesis. Together, these results indicate that C-1311 warrants further development for the treatment of PCa.

## 1. Introduction

Prostate cancer (PCa) remains one of the most common cancers in the male population and has a poor prognosis. Recent epidemiological data show that PCa is second only to lung cancer as a cause of cancer-related death among males in the United State [1]. Unfortunately, the 5-year survival rate of advanced PCa is 29% [2]. PCa is a highly heterogeneous disease, at both the molecular and cellular level, with a complex pattern of metastatic spread [3]. Such heterogeneity underlies the mixed response of PCa cells to treatment, highlighting the need for multi-targeted therapies.

The androgen receptor (AR) is a key transcription factor that drives PCa tumorigenesis and progression. Accordingly, androgen deprivation therapy (ADT), which targets the AR via chronic administration of gonadotropin-releasing hormone analogs, anti-androgens, or their combination, is considered a standard therapy for patients with locally advanced or metastatic PCa [4]. However, despite initial response rates in excess of 80%, patients invariably develop resistance to this therapy within 2–3 years, transitioning to a more aggressive form of PCa due to adaptive mechanisms or clonal selections [5,6]. Advanced tumors, often termed metastatic castration-resistant PCa (mCRPC), may still have abnormally activated AR signaling pathways that fuel tumor survival and proliferation even in the presence of ADT [7]. In this context, novel therapeutic agents that target the AR are urgently required.

The 1-nitroacridines are potent DNA-binding compounds that inhibit the growth of tumor cells [8], and have shown promising pre-clinical activity in colon [9] and prostate cancer cell lines [10]. The 1-nitroacridine, C-1748, has been previously reported to block AR activity in hormone-dependent PCa models in vitro and inhibit the growth of PCa xenografts in mice [8]. Despite those promising pre-clinical results, C-1748 has not progressed to clinical trial. In an effort to improve the efficacy and toxicity profile, a second generation of acridine derivatives, the imidazoacridinones, has been developed. The most active imidazoacridinone, C-1311 (Symadex), was well tolerated in Phase I clinical trials in patients with advanced solid tumors [11], and demonstrated promising efficacy in Phase II studies in patients with breast cancers refractory to anthracyclines and taxanes [12]. C-1311 is a DNA-damaging intercalator and inhibitor of topoisomerase II activity [13], which also blocks angiogenesis by targeting hypoxia-inducible factor 1α (HIF-1α) [14]. Additionally, C-1311 inhibits the oncogenic receptor tyrosine kinase FLT3 [15], and sensitizes p53-mutated cancer cells to radiation [16]. However, its efficacy in PCa cells never has been determined. Imidazoacridinones contain an imidazole ring attached to an acridinone core and diaminoalkyl side chain, similar to that found in topoisomerase II inhibitor, mitoxantrone. Given that mitoxantrone in combination with cabazitaxel has recently shown durable responses in patients with mCRPC [17], we investigated the anti-prostate cancer potential of the imidazoacridinones. Here, we show for the first time that imidazoacridinone C-1311, in addition to its known DNA-damaging properties, is a promising agent that blocks the transcriptional activity of the AR and effectively kills PCa cells, irrespective of their AR status.

## 2. Materials and Methods

### 2.1. Chemicals and Reagents

The imidazo- and triazoloacridinones were synthesized at the Department of Pharmaceutical Technology and Biochemistry, at the Gdansk University of Technology (Poland), as described previously [18,19], and were a kind gift from the late Prof. Jerzy Konopa. Drugs were stored at −20 °C as 50 mM stock solutions in 50% (*v*/*v*) ethanol, and freshly diluted in water before use. All other chemicals, unless stated otherwise, were purchased from Sigma-Aldrich (Sigma-Aldrich, Darmstadt, Germany).

### 2.2. Cell Lines and 3D Spheroid Cultures

The human prostate cancer LNCaP (ATCC^®^ CRL-1740^™^) and DU-145 (ATCC^®^ HTB-81D^™^) cell lines were purchased from American Type Culture Collection (ATCC, Manassas, VA, USA). The 22Rv1 cells were a kind gift from Prof. Valentine Macaulay (University of Oxford). LNCaP and 22Rv1 cells were maintained in RPMI 1640 medium. DU-145 cells were grown in DMEM. All media were supplemented with 10% FBS (Sigma-Aldrich, Darmstadt, Germany), 100 U/mL penicillin, and 100 µg/mL streptomycin (Sigma-Aldrich, Darmstadt, Germany). Cells were kept at 37 °C under a 5% CO_2_. Cells were routinely tested for mycoplasma and found to be negative. For spheroid formation, 5000 cells were seeded in 200 μL of complete growth medium in round bottom, ultra-low attachment 96-well plates (#7007, Corning, New York, NY, USA). Spheroids were grown for 5–10 days, after which the 22Rv1 and DU-145 cells produced spheroids of approx. 400 μM in diameter, while the LNCaP cells yielded much larger spheroids (approx. 800 μM in diameter). The medium was renewed, and the spheroids were subsequently treated with the drug for 96 h. Spheroid size was monitored using the GelCount imaging system (Oxford Optronix).

### 2.3. Plasmid Construction

AR reporter vector (AREIII)_3_-(AREII)_3_-(AREI)_3_-tk-Luc was generated by insertion of three copies of the chemically synthesized ARE sequence from the *PSA* gene and tk (tymidine kinase) promoter into a pGL4hygro vector (Promega, Madison, WI, USA). The three ARE sequences were as follows: AREIII (5′-TCGACGAGGAACATATTGTATCGAG-3′) [20], AREII (5′-TCGACAGGGATCAGGGAGTCTCACCAGGGATCA-3′) [21] and AREI (5′-GATCCTTGCAGAACAGCAAGTGCTAGCTG-3′) [22].

### 2.4. Generation of the LNCaP-ARE-Luc Reporter Cell Line

LNCaP cells were seeded on 6-well plates and transfected with 2 μg of the (AREIII)_3_-(AREII)_3_-(AREI)_3_-tk-Luc plasmid using the FugeneHD transfection reagent (Promega). Individual hygromycin B-resistant cell colonies were isolated, expanded, and screened for the DHT-inducible expression of luciferase. To assess the AR transactivation activity following 24 h drug treatment, cells were lysed, and the level of firefly luciferase was measured using the Luciferase Assay System (Promega), according to the manufacturer’s instructions.

### 2.5. Transient Transfection with the ARE-Luc Vector

LNCaP cells were seeded on a 96-well plate (10,000 cells per well) overnight, co-transfected with the (AREIII)3-(AREII)3-(AREI)3-tk-Luc plasmid or the pG4.14luc/hygro (Promega) and pCMV-SEAP plasmid (a kind gift from Dr. S. Schlatter) using the FugeneHD transfection reagent. Cells were treated with an experimental compound 24 h after transfection, and reporter gene activity was assayed after a further 24 h. Luciferase activities were normalized to the corresponding SEAP activity, which was used as the transfection efficiency control.

### 2.6. Cell Viability Assays

Cell viability was measured using an MTT (3-(4,5-diethylthiazol-2-yl)-2,5-diphenyltetrazolium bromide) assay. Briefly, cells (2 × 10^5^) were seeded in 24-well plates, left to attach overnight, and treated with the drugs for 72 h. After exposure, 0.5 mg/mL MTT was added into each well, and the plates were incubated for 4 h at 37 °C. The medium was removed, and the remaining formazan crystals were dissolved in DMSO. Absorbance was measured at 540 nm. The viability of the cells in the 3D spheroid cultures was measured using a resazurin assay. Briefly, single spheroids were grown in 200 µL of culture medium in 96-well plates. Following the drug treatment, 100 µL of the medium was removed, and replaced with a 20 μL resazurin solution (0.15 mg/mL) in PBS. After incubation for 4 h at 37 °C, the absorbance was measured at an excitation/emission wavelength of 544/590 nm. Cell viability was expressed as the percentage of the vehicle-treated control and the cytotoxic effect of the drug was assessed by the IC_50_ values.

### 2.7. Western Blot Analysis

Following the drug treatment, cells were lysed for 20 min on ice with buffer containing 50 mM Tris-HCl (pH 7.4), 5 mM EDTA 1% Nonidet P-40, 150 mM NaCl, 0.1% SDS, 0.5% sodium deoxycholate, 50 mM NaF, 50 mM β-glycerolophosphate, 1 mM PMSF, 1 mM sodium orthovanadate, and a protease inhibitor cocktail (Roche Applied Science, Basel, Switzerland). Proteins (50 μg) were separated by SDS-PAGE and transferred to PVDF membranes. The following antibodies were used: anti-PSA (#5877 Cell Signaling Technology, Danvers, MA, USA), anti-AR (#5153S Cell Signaling Technology), anti-GAPDH-HRP conjugate (#8884S Cell Signaling Technology), anti-γH2AX (#05-636-I, Millipore, Burlington, MA, USA), #anti-p53 (DO-I, Santa Cruz Biotechnology, Dallas, TX, USA), anti-p21 (#2947 Cell Signaling Technology), anti-PARP (#9542 Cell Signaling Technology), and anti-β-actin (C4, Santa Cruz Biotechnology, Dallas, TX, USA). Immunoblots were developed using the Li-COR Odyssey imaging system, except for the anti-PSA, anti-AR, and anti-GAPDH-HRP conjugate, which were visualized using an enhanced chemiluminescence detection kit, the SuperSignal West Pico Chemiluminescent Substrate (Thermo Fisher Scientific, Waltham, MA, USA).

### 2.8. ChIP Assay for the Androgen Receptor

LNCaP cells were cultured until an 80% confluence and exposed to C-1311. Proteins were cross-linked with formaldehyde, and then the cells were harvested, lysed, and the DNA sonicated with a VCX-130 sonicator (Sonics & Materials Inc., Newtown, CT, USA). Chromatin immunoprecipitation was performed using the Magna ChIP™ A/G Chromatin Immunoprecipitation Kit (EMD Millipore, Burlington, MA, USA) according to the manufacturer’s protocol. The following antibodies were used: control mouse IgG (EMD Millipore, Burlington, MA, USA) and anti-androgen receptor antibody—ChIP Grade (#ab74272, Abcam, City, UK). The relative enrichment of the *PSA* gene sequences was analyzed with real-time PCR using the SYBR Green I Master Mix on a LightCycler 96 (Roche, Basel, Switzerland). Primers P1 complementary to the *PSA* were as follows: forward 5′-GAGTGCTGGTGTCTTAGGGC-3′ and reverse 5′-GCTAGCACTTGCTGTTCTGC-3′; primers P2 complementary to the PSA intron 1 were as follows: forward 5′-CCTCTTCCAGCAACTGAACC-3′ and reverse 5′-TCAGGGTTGACAGGAGGAAC-3′. The AR negative-binding region in the first intron of the PSA was identified using Matinspector software [23]. The amplification of the soluble chromatin prior to immunoprecipitation was used as an input control. Quantification was performed using the dCt method with the Ct obtained for input DNA as a reference value: 1000 × 2-dCt, where dCt = Ct sample − Ct input DNA.

### 2.9. Quantitative Real-Time Polymerase Chain Reaction (RT-qPCR)

Total RNA was purified from LNCaP and DU-145 cells with the mirVana miRNA Isolation Kit (Thermo Fisher Scientific, Waltham, MA, USA) according to the manufacturer’s instruction. The RNA concentration, purity, and integrity were assessed by Qubit (Invitrogen, Carslbad, CA, USA) and Tape Station 2200 (Agilent Technologies, Santa Clara, CA, USA). cDNA was synthesized using the Transcriptor High Fidelity cDNA Synthesis Kit (Roche, Basel, Switzerland) according to the manufacturer’s protocol. The mRNA expression levels were measured using RT-qPCR performed in 20 µL volumes containing SYBR Green I Dye using the LightCycler^®^ 480 SYBR Green I Master (Roche, Basel, Switzerland). The sequences of the primers were as follows: *AR* forward, 5′-GGAATTCCTGTGCATGAAA-3′; *AR* reverse, 5′-CGAAGTTCATCAAAGAATT-3′ [24]; *GAPDH* forward, 5′-TGCACCACCAACTGCTTAGC-3′; *GAPDH* reverse, 5′-GGCATGGACTGTGGTCATGAG-3′ [25]. The levels of *AR* were normalized to the *GAPDH* values. The quantitative PCR values were determined for each of the mRNAs levels using the comparative Ct (ΔΔCt) method.

### 2.10. Flow Cytometry Analysis

Following drug treatment, BrdU (20 µM) was added and the cells were incubated for 1 h in dark before fixation in ice-cold 70% ethanol. Samples were treated with 2 M HCl and blocked in 2% FBS in PBS solution before treatment with anti-BrdU primary antibody (BD Biosciences, San Jose, CA, USA) and secondary Alexa Fluoro 488 antibody (Invitrogen). Propidium iodide (Sigma Aldrich, Darmstadt, Germany) was added to the samples to determine the total DNA content.

For the detection of apoptosis, cells, following drug treatment, were stained using the Annexin V-FITC Apoptosis Detection Kit (#556547, BD Pharmingen, San Jose, CA, USA) according to the manufacturer’s instructions. Samples were run on a BD FACSCalibur machine (BD Biosciences, San Jose, CA, USA) and plots were analyzed with FloJo software (v10, LLC, Ashland, OR, USA).

### 2.11. RNA-Seq Sample Preparation and Sequencing

RNA-seq libraries were constructed from 1 µg of total RNA with an RNA integrity number (RIN) > 8, using the Illumina TruSeq^®^ Stranded Total RNA Library Prep Kit (#20020598, Illumina, San Diego, CA, USA). Indexed libraries were pooled, clustered with use the cBot, and sequenced on the Illumina HiSeq 4000 platform generating 150 bp paired end reads (2 × 75 bp). Sequencing data were processed to obtain fastq files with the bcl2fastq pipeline (Illumina, San Diego, CA, USA), including demultiplexing and adapter trimming steps. The quality of the reads was assessed using FastQC (Babraham Institute, Cambridge, UK). The BBduk tool (U.S. Department of Energy Joint Genome Institute, Walnut Creek, CA, USA) was used to filter and soft trim all reads of high quality, as well as to filter out fragments originated from rRNAs. Cleaned reads were mapped to the human reference genome GRCh38 using the splice-aware aligner STAR [26]. Unique counts per gene were calculated with the in-built option in STAR and used for data visualization and further differential gene expression analysis. The quality of the mapping was assessed with the Flagstat module from Samtools [27] as well as RSeQC [28]. Mapped reads were also visually inspected with the Integrative Genomics Viewer [29]. Samples size factors were estimated, and counts were normalized using the trimmed mean of the M-values (TMM). Differential expression analysis was performed with EdgeR software [30], assuming a negative binomial distribution. After model fitting, the dispersion estimates were obtained, and a general linearized model was applied. The likelihood ratio test (LRT) was used to call differentially expressed genes (DEGs) under |log2FC| > 1 and a false discovery rate (FDR) corrected *p*-value < 0.05. For functional analysis, the gene ontology (GO) enrichment analysis—biological process (BP) by STRING (https://string-db.org) and the Ingenuity Pathway Analysis (QIAGEN Inc., https://www.qiagenbioinformatics.com/products/ingenuity-pathway-analysis) were used. The significance of the association between the data set and the canonical pathway was determined based on two parameters: (a) the ratio of the number of genes from the data set that map to the pathway divided by the total number of genes that map to the canonical pathway; and (b) a *p* value calculated using Fischer’s exact test determining the probability that the association between the genes in the data set and the canonical pathways is due to chance alone. The RNA-seq data were deposited in the Gene Expression Omnibus (GEO) database (accession number: GSE151113).

### 2.12. Statistical Analysis

Statistical differences were determined using GraphPad Prism 8 software (v8.4.3, GraphPad Software, Inc., San Diego, CA, USA). Unless otherwise indicated, a one-way ANOVA test was used, followed by a Dunnett test for each comparison. A *p* value less than 0.05 was considered significant (* *p* < 0.05; ** *p* < 0.01; *** *p* < 0.001; **** *p* < 0.0001). Gene Set Enrichment Analysis (GSEA) was performed using the WebGestaltR R package with a ranked list of all the genes based on log10 (FDR-corrected *p*-value) and a change direction as up- (+) or downregulated (−). Over-representation analysis of the ‘AR targets upregulated by AR’ was performed with a hypergeometric test using the clusterProfiler R package and applying as the background the list of genes, which were analyzed in RNA-seq. Volcano plots were created in the R environment. Heat maps were generated with the “pheatmap” R package and GraphPad Prism 8. Venn diagrams were created with BioVinci software (v1.1.5, BioTuring, San Diego, CA, USA).

## 3. Results

### 3.1. Imidazoacridinones Are Potent Against AR-Dependent PCa Cells

**Figure 1:**
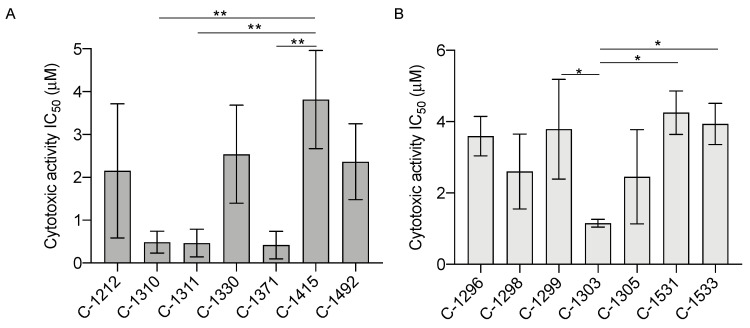
Cytotoxic activity of the (**A**) imidazoacridinones and (**B**) triazoloacridinones in AR-dependent prostate cancer LNCaP cells. IC_50_ values were determined after 72 h of treatment based on the dose–response curves and presented as bar graphs. Data are the mean ± SD, *n* = 3. Significance: One-way ANOVA with Tukey’s multiple comparison test; ** *p* < 0.01, * *p* < 0.05.

To begin, we examined the in vitro cytotoxic activity of the imidazoacridinones (Figure S1A) and their close analogs, the triazoloacridinones (Figure S1B), which contain a triazole ring condensed with the acridinone chromophore. The set of seven imidazoacridinones and triazoloacridinones, with diverse chemical structures, were screened for their cytotoxic effect on AR-dependent LNCaP cells. Cells were incubated with the drugs for 72 h and the cell viability was determined using an MTT assay. The resulting IC_50_ values indicated that the LNCaP cells were particularly sensitive to three of the imidazoacridinones, C-1310, C-1311, and C-1371 (Figure 1A and Figure S2A).

These active compounds share structural features, including the presence of a diaminoethyl side chain and hydroxyl group attached to an acridinone ring (Figure S1A). Interestingly, among the triazoloacridinones, only the C-1303 derivative, which is a structural analogue of imidazoacridinone C-1311, had a significantly lower IC_50_ value (Figure 1B and Figure S2B). Together, these data suggest that the selected imidazoacridinones may be effective against PCa cells. As C-1311 has already demonstrated limited toxicity and high efficacy in Phase II clinical trials, we focused further studies on this compound.

### 3.2. C-1311 Induces Global Differences in the Transcriptome Profiles of PCa Subtypes

**Figure 2:**
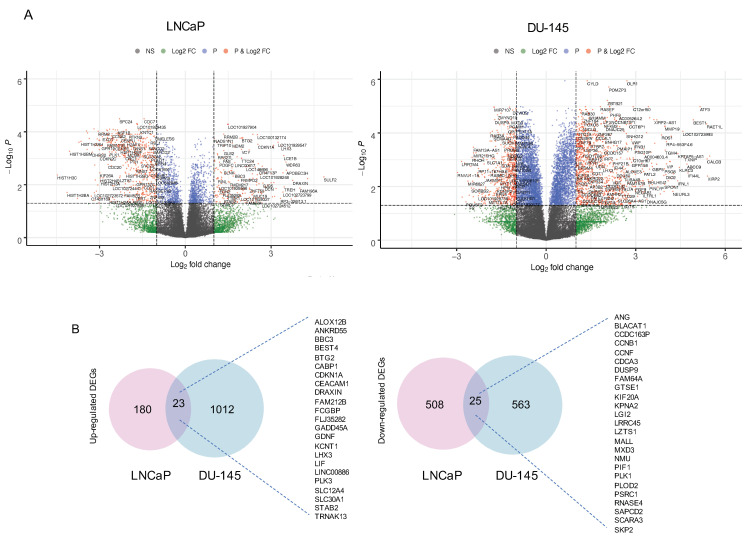
Global changes in the C-1311-regulated transcriptional profile in PCa cells. LNCaP and DU-145 cells were treated with 1 µM C-1311 for 24 h and subjected to RNA-seq analysis (*n* = 3). (**A**) Volcano plot of the differently expressed genes in PCa cells exposed to C-1311. The horizontal line at a false discovery rate (FDR) = 0.05; vertical line at |log2FC| = 1. (**B**) Venn diagram of differentially expressed genes (DEGs) in PCa cells exposed to C-1311.

The AR acts as a ligand-regulated transcription factor [31]. Given the potent activity of the selected imidazoacridinones in the AR-positive LNCaP cells, next we examined how these compounds affect global gene expression in PCa cells with a different AR status. To this end, we performed transcriptomic profiling using next-generation sequencing (RNA-seq) for one of the most active imidazoacridinones, C-1311. Differentially expressed genes (DEGs) were determined based on the FDR < 0.05 and |log2FC| > 1. As shown by the volcano plots (Figure 2A) and Venn diagram (Figure 2B), C-1311 induced significant changes in the whole transcriptome signature of the PCa cells. In AR-positive LNCaP cells, 203 genes were upregulated and 533 genes were downregulated, whereas in AR-negative DU-145 cells, 1035 genes were upregulated and 588 genes were downregulated. Among the up- and downregulated genes, only 23 (1.9%) and 25 (2.2%), respectively, overlapped between LNCaP and DU-145, which was consistent with the different AR-dependency of these two PCa cell lines.

Next, we analyzed the functional categories and canonical pathways for the DEGs using STRING (https://string-db.org) and the Ingenuity Pathway Analysis (IPA; QIAGEN Inc., https://www.qiagenbioinformatics.com/products/ingenuity-pathway-analysis), respectively. As shown by the GO BP enrichment analysis, the C-1311 treatment in AR-positive LNCaP cells induced significant changes in expression of genes involved in the DNA damage response, cell division, and cell cycle-related pathways (mitotic cell cycle, chromosome organization) (Figure S3A). A detailed IPA analysis of the altered canonical pathways showed the most significant gene transcript enrichment in the pathways was related to the G2/M DNA damage checkpoint regulation, ATM signaling, BRCA1-mediated DNA damage response, or cell cycle control of replication (Figure S3B and Table S1). Surprisingly, BP gene ontology enrichment revealed that in AR-negative DU-145 cells treated with C-1311, the DEGs were mainly involved in the regulation of metabolic processes (Figure S3A); in particular, glycolysis, gluconeogenesis, or glucocorticoid receptor signaling (Figure S3B and Table S2). Together, these results indicate that the transcriptional response of PCa cells to C-1311 is heterogeneous.

### 3.3. C-1311 Significantly Blocks Androgen Receptor Transactivation Activity

In AR-positive LNCaP cells treated with C-1311, the DEGs in BP were mainly enriched in processes regulating cell cycle progression. Several studies show that AR signaling is significantly implicated in DNA replication, checkpoint mechanisms, cell division, or DNA damage response, and inhibition of AR activity may lead to the induction of severe DNA damage and apoptosis of PCa cells [32,33].

**Figure 3:**
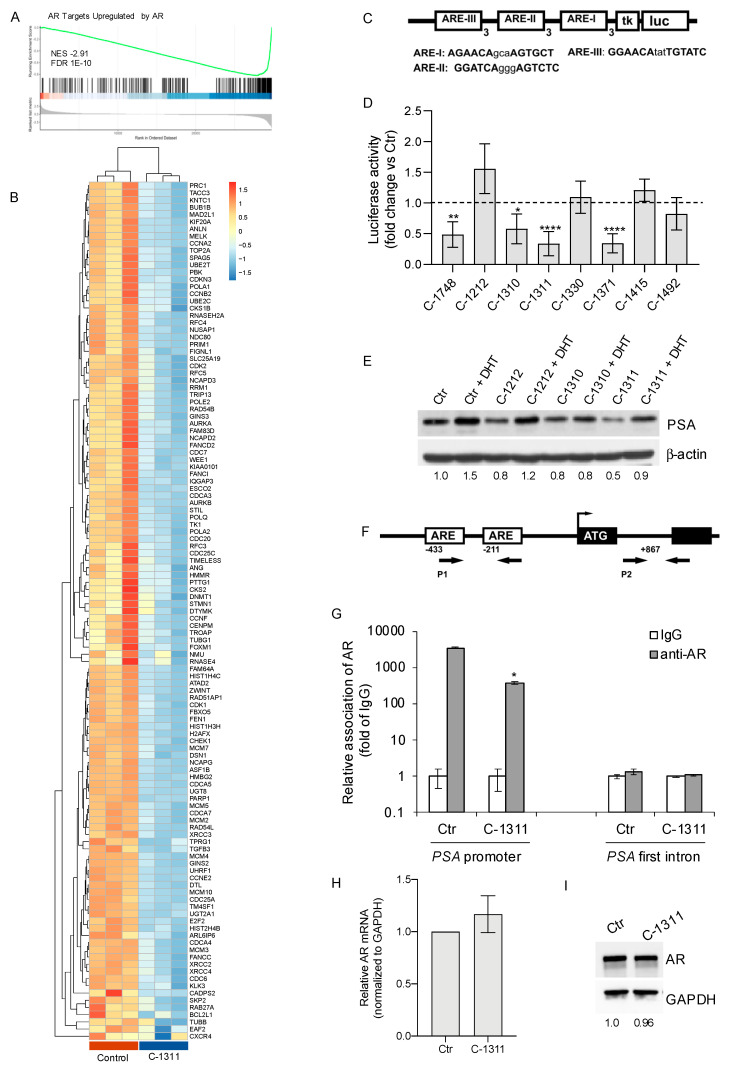
C-1311 blocks transcriptional activity of the androgen receptor (AR). (**A**) Gene Set Enrichment Analysis (GSEA) plot showing repression of the ‘AR Targets Upregulated by the AR’ gene signature in LNCaP cells treated with 1 µM C-1311 for 24 h. (**B**) Heat map of DEGs (|log2FC| > 1, FDR < 0.05) identified from the ‘AR Targets Upregulates by AR’ gene signature in LNCaP cells treated with 1 µM C-1311 for 24 h. (**C**) Schematic representation of the ARE-Luc reporter vector utilized for generation of the LNCaP-ARE-Luc reporter cell line. The ARE-Luc vector contains three copies of each of the three ARE sequences derived from the *PSA* gene and tk (thymidine kinase) basal promoter. *Luc: luciferase* gene. (**D**) AR transactivation assay. LNCaP-ARE-Luc cells were treated with imidazoacridinones at 1 µM for 24 h. Nitroacridine C-1748 (0.01 µM) was used as a reference AR inhibitor [8]. Luciferase activity (fold-change as compared to untreated cells) is shown. Data are the mean ± SD, *n* = 8. Significance: One-way ANOVA test; **** *p* < 0.0001, ** *p* < 0.01, * *p* < 0.05. (**E**) Effect of selected imidazoacridinones on the expression of prostate-specific antigen (PSA). LNCaP cells were exposed to 5 µM imidazoacridinone or pre-incubated with 10 nM 5-α-dihydrotestosterone (DHT) to potentiate the transcriptional activity of the AR. The level of PSA was determined 24 h later by Western blotting. Bands were analyzed by densitometry using ImageJ software and normalized to β-actin. Numbers represent a fold change in the PSA level relative to the untreated control cells not stimulated with DHT. (**F**–**G**) C-1311 blocks the recruitment of AR to ARE in the *PSA* promoter. LNCaP cells were treated with 5 µM C-1311 for 8 h followed by ChIP analyses with a control IgG and anti-AR antibody. (**F**) Schematic diagram showing the position of the AREs located in the proximal *PSA* promoter. P1 and P2: Locations of the PCR primers used in ChIP-qPCR. ATG: Start codon. (**G**) The effect of C-1311 on the recruitment of AR to the ARE located in the proximal promoter of the *PSA* and to the sequences of the first intron of the *PSA*, as indicated in (**F**). Binding of AR is shown as the fold enrichment over a normal IgG. Data are the mean ± SD of quadruplicates RT-PCR analyses from a representative ChIP experiment. Significance: Student’s *t*-test; * *p* < 0.05 in comparison to the control. (**H**) Real-time PCR analysis of the AR mRNA level in the LNCaP cells exposed to 1 µM C-1311 for 24 h. Data are the mean ± SD, *n* = 2. (**I**) Western blotting analysis of AR protein levels in LNCaP treated as in (**H**). Numbers represent the fold change in the AR level normalized to GAPDH and expressed relative to the control cells.

Thus, next we focused on the effect of C-1311 on the AR-mediated transcriptional response. For this purpose, we designed the ‘AR targets upregulated by the AR’ gene signature (Table S3), using the TRRUST v2 database (Transcriptional Regulatory Relationships Unraveled by Sentence-based Text mining) (https://www.grnpedia.org/trrust/) [34] and recurrent AR target genes in the literature [32,35,36,37]. RNA-seq data were interrogated for the enrichment in the tested AR target gene signature. Gene Set Enrichment Analysis (GSEA) indicated the strong repression of the ‘AR targets upregulated by the AR’ gene signature (NES = −2.91, FDR = 1 × 10^−10^) (Figure 3A). Detailed analysis showed that of the 409-gene ‘AR targets upregulated by the AR’ signature, 117 genes identified as DEGs, were significantly repressed by C-1311 (over-representation analysis, Fisher’s exact test *p*.adjust = 4.58 × 10^−99^). For example, C-1311 decreased the expression of the AR target genes that are known to regulate DNA repair (PARP1, RAD54L), control of the G2/M phase progression (CDC6, CDC20, CDK1, CCNA2, CCNB2, CDK2), mitotic division, spindle checkpoint, and cytokinesis (MAD2L1, NUSAP1, PRC1, KIF20A, ZWINT) (Figure 3B).

RNA-seq analysis suggested that C-1311 may block the function of the AR through the repression of its downstream transcriptional targets. To further test this, we generated a stable reporter cell line (LNCaP-ARE-Luc) expressing luciferase under the control of the tk promoter containing ARE (androgen response element) sequences derived from the prostate-specific antigen (PSA) promoter (Figure 3C). LNCaP-ARE-Luc cells were treated and screened for AR-mediated luciferase activity. C-1311, as well as other imidazoacridinones like C-1310 and C-1371, at a 1 µM concentration significantly reduced the luciferase activity to a level lower than 50% of that observed in the untreated cells (Figure 3D). C-1311 and C-1371 were also effective at ten-fold lower doses (0.1 µM, Figure S4A), indicating a dose-dependent inhibition of the transcriptional activity of the AR. Importantly, additional experiments using LNCaP cells transiently transfected with an empty vector or ARE-Luc reporter vector, confirmed that C-1311 significantly decreased the ARE-responsive luciferase activity, while it had no effect on the empty control vector (Figure S4B). The triazoloacridinones did not significantly inhibit AR transactivation activity (Figure S4C). Next, we confirmed the anti-AR activity of the selected imidazoacridinones by measuring the level of PSA. PSA expression is regulated by an upstream promoter and enhancer androgen response elements (ARE). Elevated PSA levels are a widely used marker to demonstrate activation of the AR signaling pathway [38]. Western blot analysis showed that imidazoacridinone C-1311 reduced the level of PSA in LNCaP cells. This inhibitory effect was still evident when AR was additionally stimulated using 5-alpha-dihydrotestosterone (DHT) (Figure 3E). The C-1311-mediated decrease in AR transcriptional activity was further confirmed by a chromatin immunoprecipitation (ChIP) assay (Figure 3G). C-1311 significantly reduced binding of AR to the AR response element (ARE) of the PSA promoter in LNCaP cells. Importantly, we did not observe the recruitment of AR to the AR-negative binding region located in the first intron of the *PSA*, in either the control or C-1311-treated cells (Figure 3G). In addition, RT-PCR and Western blotting analyses showed no significant changes in the AR mRNA (Figure 3H) and protein (Figure 3I) level following C-1311 exposure.

Together, these data indicate that the selected imidazoacridinones, including the lead compound C-1311, are promising inhibitors of AR activity. Mechanistically, C-1311 interferes with AR binding to the ARE and blocks the AR-mediated transcription.

### 3.4. C-1311 Is Also Potent against AR-Independent PCa Cells in 2D Monolayer and 3D Spheroid Cultures

**Figure 4:**
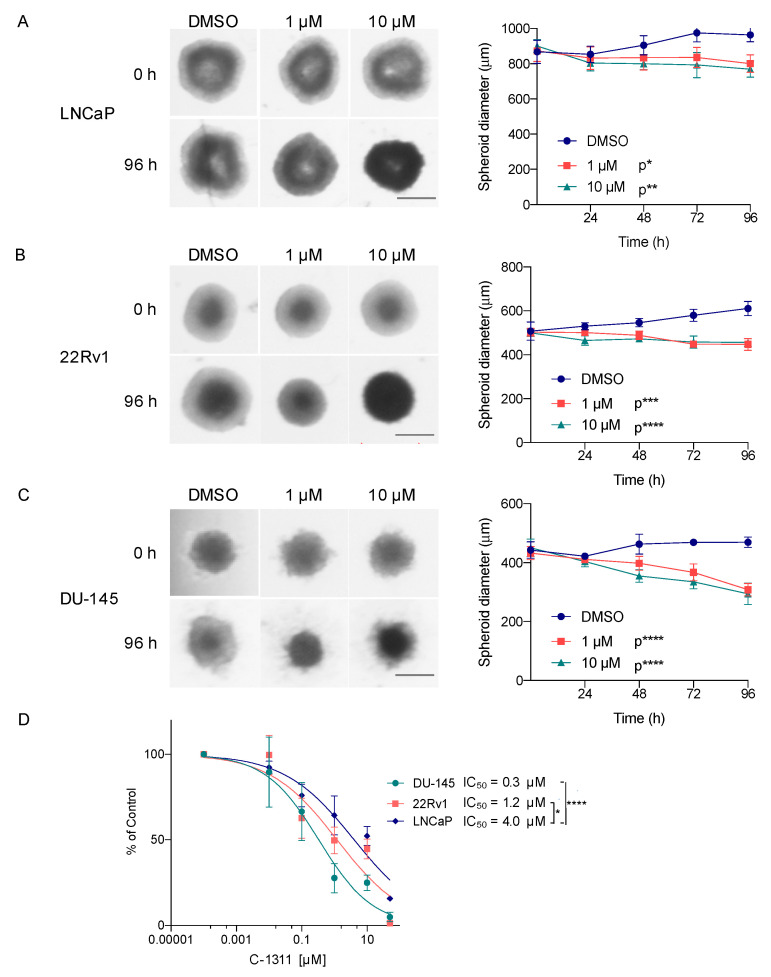
C-1311 is also potent against the AR-independent PCa cells in 3D spheroid cultures. (**A–C**) Representative images of the PCa cell spheroids treated with DMSO or C-1311 at the indicated doses for 96 h. Scale bars = 500 µm. Right panels show changes in spheroid diameter monitored over 96 h of C-1311 exposure. Results are the mean ± SD, *n* = 4. Significance: Two-way ANOVA; **** *p* < 0.0001, *** *p* < 0.001, ** *p* < 0.01, * *p* < 0.05 in comparison to DMSO. For graph clarity, the corresponding *p*-values are presented next to the treatment conditions. (**D**) Effect of C-1311 on viability of the PCa cells grown as multicellular spheroids. Spheroids were treated with C-1311 for 96 h and the cell viability was determined by a resazurin assay. Results are the mean ± SD, *n* = 4. Significance: Two-way ANOVA; **** *p* < 0.0001, * *p* < 0.05.

Given that C-1311 is a multi-target therapeutic, we expanded our analysis of its effects on PCa cell lines to include those with a differing AR status. As expected, C-1311 did not significantly change expression of the androgen-regulated genes in AR-negative DU-145 cells (Figure S5). Surprisingly, however, C-1311, as well as other imidazoacridinones, also inhibited the growth of DU-145 cells (Figures S6A,B), and this effect was more pronounced compared to the LNCaP cells (Figure S5A vs. Figure 1A). The C-1311 IC_50_ value for the DU-145 cells was 0.15 µM, while for the LNCaP cells it was 0.47 µM. To further confirm the efficacy of C-1311 in a more physiologically relevant tumor model, we grew multicellular PCa spheroids in the presence of C-1311 or DMSO. C-1311 treatment significantly decreased the diameter of the LNCaP and DU-145 cell spheroids in a time-dependent manner (Figure 4A–C). In addition, C-1311 effectively blocked the growth of the spheroids formed from 22Rv1 cells. These cells have partial androgen sensitivity due to the expression of both a full-length AR and a constitutively active AR splice variant, AR-V7, which lacks the ligand-binding domain [39]. As such, the 22Rv1 cells serve as a model for a transition phenotype between that of androgen-sensitive LNCaP and AR-negative DU-145 cells [39].

Importantly, as shown by the resazurin assay (Figure 4D), C-1311 also significantly reduced viability of the PCa cells grown as spheroids. Again, DU-145 cells were the most sensitive to C-1311 treatment. The IC_50_ value for the DU-145 cells was 0.3 µM, whereas for 22Rv1 and LNCaP it was 1.2 and 4 µM, respectively. Numerous intrinsic differences between DU-145 and LNCaP or 22Rv1 cells may account for this difference in sensitivity. Nonetheless, these results indicate that C-1311 extends its activity also to the AR-independent PCa cells.

### 3.5. C-1311 Induces DNA Damage and Arrests Cell Cycle Progression

**Figure 5:**
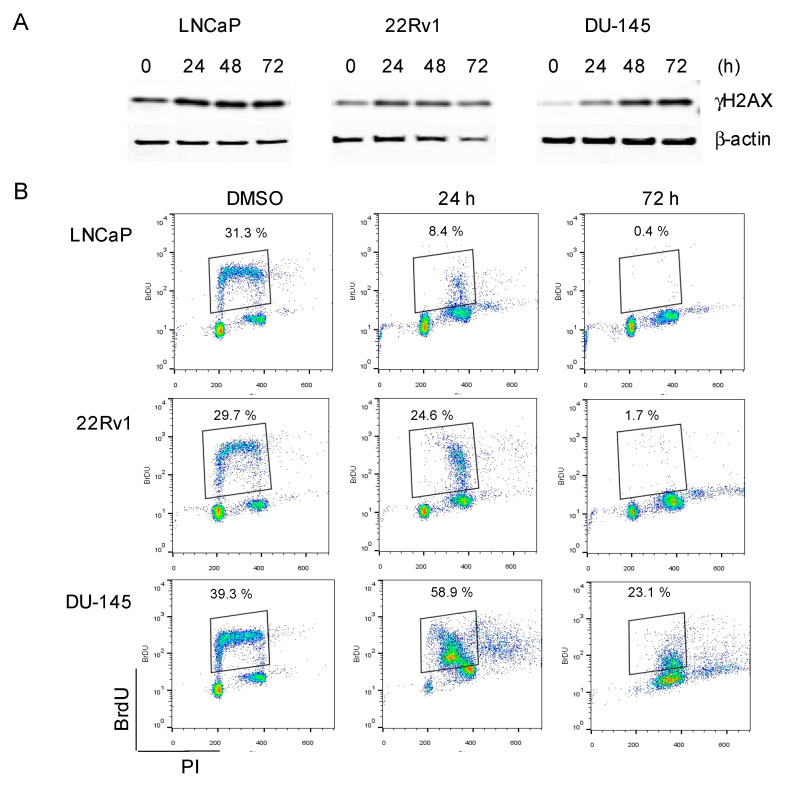
C-1311 induces DNA damage and decreases the percentage of replicating PCa cells irrespectively of AR status. (**A**) PCa cells were treated with 1 µM C-1311 for the times indicated. Western blotting was carried for γH2AX. β-actin was used as the loading control, *n* = 3. (**B**) Flow cytometry analysis of the BrdU incorporation (DNA synthesis) in PCa cells treated with C-1311 as in (**A**). Boxed region on the dot plots represents the BrdU-positive cells. Results are representative of three independent experiments and are presented as FloJo dot-plot graphs, which heat maps fluorescence intensities of greatest frequency in red.

Next, we assessed the effect of C-1311 on the biological response of the PCa cells. C-1311, as a topoisomerase II inhibitor, has been previously demonstrated to induce DNA damage [16]. Correspondingly, we found that C-1311 treatment in LNCaP, 22Rv1, and DU-145 cells increased the phosphorylation of the histone H2AX (γH2AX) (Figure 5A), a well-known marker of DNA damage [40]. In addition, as shown by the BrdU incorporation assay, C-1311, within 72 h of treatment, significantly reduced the percentage of cells actively replicating DNA (Figure 5B). In LNCaP cells, a decrease in the BrdU-positive cell fraction was already evident within 24 h of treatment, while in 22Rv1 and DU-145 cells, a 72 h treatment was required to achieve a similar effect.

**Figure 6:**
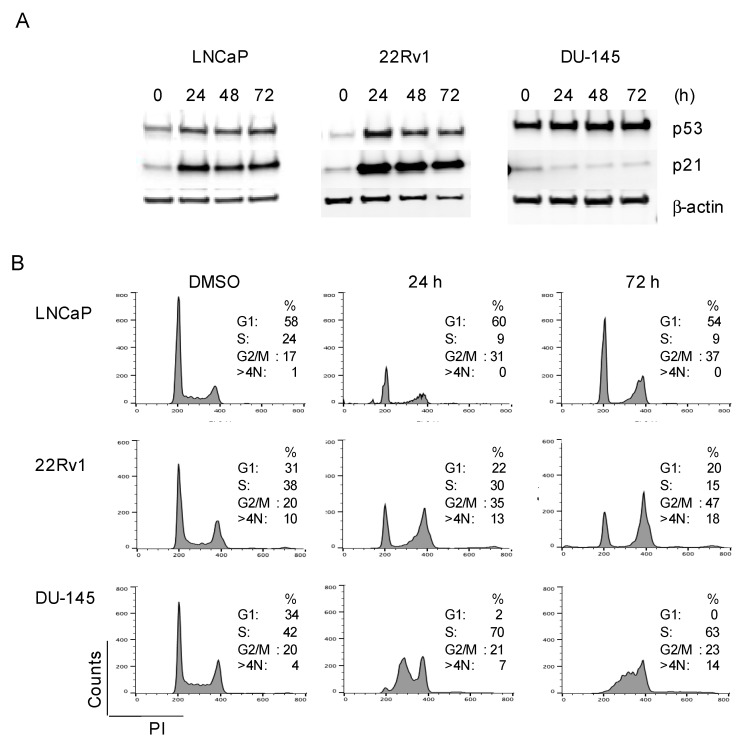
C-1311 blocks PCa cell cycle progression. (**A**) Western blotting analysis of p53 and p21 in PCa cells treated with 1 µM C-1311 for the times indicated. β-actin was used as the loading control, *n* = 3. (**B**) Cell cycle profiles of the PCa cells treated as in (**A**), stained with PI and analysed by flow cytometry. Histograms are representative of three independent experiments.

Given that C-1311 triggered DNA damage and decreased the population of replicating cells, next we examined its effect on the cell cycle progression of the PCa cells. First, we determined the level of p53 and its transcriptional target, p21, which control the G1/S and G2/M phase cell cycle checkpoints. LNCaP cells contain the wild-type p53 gene. The 22Rv1 cells harbor one wild-type copy of p53 and one mutated copy of p53 (p53 wt/Q331R) and still retain p53 transcriptional activity. DU145 cells have inactivating p53 mutations (P223L and V274F mutations) [41]. Western blot analysis showed that in LNCaP and 22Rv1 cells, C-1311 treatment increased the level of p53 and p21 (Figure 6A). In DU-145 cells, consistent with the non-functional p53, C-1311 did not increase the expression of p21. The cell cycle analysis showed that in response to C-1311, approximately 50% of the LNCaP cells remained in the G1 phase and failed to enter the S phase. In addition, C-1311 also induced arrest of the cell cycle in the G2/M phase (Figure 6B and Figure S7). The 22Rv1 cells progressively accumulated in the G2/M phase, although approximately 20% of the cells remained arrested in the G1 phase after 72 h of treatment. In DU-145 cells, lacking a p53/p21 signaling axis, a population of cells in the G1 phase disappeared, and cells, after transient accumulation in the S phase, arrested in the G2/M phase. In contrast to LNCaP cells, in the 22Rv1 and DU-145 cells, the increase in the G2/M population was also accompanied by the appearance of polyploid cells (>4N DNA). These results are consistent with studies showing that in cancer cells carrying *p53* mutations, DNA damage and a prolonged G2/M phase arrest can also lead to polyploidy [42]. Together, these results indicate that C-1311, in addition to targeting the AR function, induces DNA damage and effectively blocks PCa cell cycle progression, irrespective of AR status.

### 3.6. C-1311 Induces Apoptosis Irrespective of AR Status

Next, we examined the cellular consequences of the C-1311 treatment in PCa cells. GO analysis revealed that several DEGs identified in the LNCaP and DU-145 cells exposed to C-1311 were also enriched in apoptosis-related pathways (Tables S4 and S5). In particular, we identified 93 DEGs in the LNCaP cells and 153 DEGs in the DU-145 cells, which were associated with positive and negative regulation of apoptosis. For example, C-1311 decreased the expression of classical anti-apoptosis genes, such as the IAP family member *BIRC5* (*survivin*) or *Bcl-2*, but also *BRCA1* and *BRCA2*, which connect DNA damage and stress response pathways to the execution of cell cycle arrest and apoptosis [43].

**Figure 7:**
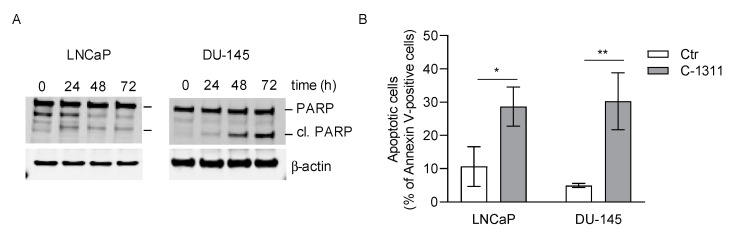
C-1311 induces apoptosis irrespective of AR status. (**A**) Western blotting analysis of apoptosis-related PARP cleavage. PCa cells were treated with DMSO or 1 µM C-1311 for the time indicated. β-actin was used as the loading control, *n* = 3. (**B**) The percentage of apoptotic cells determined by flow cytometry analysis of Annexin V/propidium iodide staining (PI). PCa cells were treated with DMSO or 1 µM C-1311 for 72 h. The bar graph shows the percentage of total Annexin V-positive cells, including Annexin V+/PI− (early apoptosis) and Annexin V+/PI+ (late apoptotic cells). Results are the mean ± SD, *n* = 3. Significance: Student’s *t*-test; * *p* < 0.05; ** *p* < 0.01.

Western blot analysis confirmed that changes in the expression of the apoptosis-related genes were accompanied by those typical for apoptosis, cleavage of PARP (Figure 7A). Similarly, flow cytometric analysis of Annexin V staining of cell surface phosphatidylserine, supported the observations that C-1311 was able to induce apoptosis in both LNCaP and DU-145 cells (Figure 7B and Figure S8).

## 4. Discussion

During PCa development, both AR-independent and AR-dependent signaling mechanisms contribute to the malignant transformation of epithelial cells [44]. Here we demonstrate that the imidazoacridinone C-1311 has a potent anti-cancer activity that extends to AR-dependent and AR-independent PCa cells. Regardless of AR status, C-1311 caused DNA damage, effectively arrested cell cycle progression in the G2/M phase, and induced apoptosis. Importantly, in AR-dependent PCa cells, C-1311 was found to inhibit transcriptional activity of AR, uncovering a novel mechanism that may be relevant for its anticancer effect. Mechanistically, C-1311 decreased AR binding to the canonical AR response element (ARE) sequence in the *PSA* promoter and reduced the expression of the PSA protein. RNA sequencing revealed that C-1311 significantly downregulated the transcription of several other AR-dependent genes, confirming that C-1311 targeted AR transcriptional activity. These results provide first evidence that C-1311 interferes with the AR binding to DNA. We have previously found that C-1311 also inhibited the DNA-binding capacity of transcriptional factor HIF1 α to the hypoxia responsive element (HRE) sequence [14]. One of the key mechanistic characteristics of C-1311 is that it is a DNA intercalator. It is therefore possible that through intercalation, C-1311 may interfere with the binding of other transcription factors to their cognate sequences. In addition, given that C-1311 is also a topoisomerase II inhibitor, its non-specific effect on the global transcription cannot be ruled out. Topoisomerase II can bind within promoters of the actively transcribed genes [45], and topoisomerase II poisons, like adriamycin, were found to cause structural changes at or near the promoters and alter gene expression, although this effect depends on the arrangement of the promoter sites and the disposition of the topoisomerases [46]. While further studies are required to address these possible non-specific effects, the C-1311-mediated inhibition of the transcriptional activity of AR is a promising additional function of this drug, which may help to eliminate the androgen-dependent PCa cells.

In PCa, mutations in the *p53* tumor suppressor gene are associated with disease progression, increased metastasis, and androgen-independent growth [47]. Mechanism-of-action studies indicated that in AR-positive PCa cells expressing functional p53, C-1311 induced stable G1 and G2/M arrest. In contrast, AR-negative cells with mutated *p53* underwent arrest in the G2/M phase of the cell cycle. These results are in agreement with our previous observations showing that C-1311 has the potential to kill cancer cells with an unfavorable *p53* profile [16]. Strategies combining the standard anti-PCa therapy with cell cycle inhibitors targeting the G1 (palbociclib, ribociclib, AZD-5363, ipatasertib) or G2 (adavosertib) phases of the cell cycle are currently tested in several clinical trials in castration-resistant prostate cancer [48]. In this light, the potential of C-1311 to effectively block cell cycle progression of PCa cells in the G1 or G2/M phases is particularly promising. However, it should be noted that DNA damage and prolonged G2/M arrest can also carry the risk of abnormal mitosis and development of polyploid cells [42]. We found that in AR-independent PCa cells with mutated *p53*, C-1311-induced G2/M arrest was accompanied by the appearance of a population (~18%) of polyploid cells. While studies show that, following treatment with DNA-damaging agents, polyploid cells often undergo cell death or terminally arrest in senescence, polyploidy may also represent a survival mechanism [49,50]. Further studies are required to address the long-term fate of PCa polyploid cells arising following C-1311 exposure and their effect on the drug’s efficacy.

Interestingly, using transcriptome sequencing, we identified differentially expressed genes (DEGs) and signaling pathways that may serve as additional targets for C-1311 and contribute to its anti-PCa activity. Multiple lines of evidence link AR signaling to the DNA damage response (DDR). AR regulates a transcriptional program of DNA repair, including homologous recombination (HR), activation of the poly(ADP-ribose) polymerase (PARP) function, and non-homologous end-joining recombination (NHEJ) [20]. Accordingly, inhibition of AR has been clinically shown to sensitize PCa to DNA-damaging chemo- and radiotherapy [51]. Employing RNA-seq, we found that in AR-positive cells, C-1311 significantly decreased the transcription of *BRCA1* and *BRCA2*, two essential genes in the HR pathway. Moreover, C-1311 also decreased expression of direct AR target genes that play a functional role in HR, including *CHEK1*, *RAD54L*, *RAD54B*, *POLE2*, and *XRCC2*. Downregulation of HR in response to the inhibition of the AR by enzalutamide has been shown to sensitize PCa cells to PARP inhibitors [52]. Although it needs a separate study, this raises the possibility that C-1311, through the induction of HR deficiency, may be synthetically lethal with PARP inhibitors.

Gene ontology and network analysis of the DEGs indicated that in AR-negative DU-145 cells, in contrast to AR-positive LNCaP cells, C-1311 treatment affected the transcriptional program of cellular metabolism. Studies show that PCa progression, aggressiveness, and poor prognosis are associated with an increased glycolytic phenotype. The highly metastatic PCa consume more glucose, with consequent lactate production, compared to the poorly metastatic PCa [53]. In AR-negative PCa cells, C-1311 significantly downregulated the expression of the key glycolytic enzymes *PGK1*, *GPI*, *PPI1*, *PGAM1*, *ALDOA*, and *ALDOC*. These results suggest that C-1311, by targeting glycolysis, may promote metabolic stress in androgen-independent PCa cells. Whether the C-1311-mediated modulation of the transcriptional program of PCa cell metabolism translates into a real cytotoxic effect warrants further studies.

## 5. Conclusions

Here, we show that the anticancer imidazoacridinone C-1311 is highly effective in AR-dependent and AR-independent PCa cells. Previous studies identified C-1311 as a multitargeted compound, which blocks activity of topoisomerase II [13], HIF-1α/VEGF signalling, tumour angiogenesis [14], and FLT3 receptor tyrosine kinase FLT3 [15]. We demonstrated that, in addition to these functions, C-1311 blocks the transcriptional activity of the AR. Single drugs that affect multiple targets are considered as a valuable opportunity for drug discovery [54]. In this context, the newly identified inhibitory potential of C-1311 against the AR makes this drug a valuable candidate for further testing against prostate cancer.

## Supplementary Materials

The following are available online at https://www.mdpi.com/2227-9059/8/9/292/s1. Figure S1: Chemical structures of (A) imidazoacridinones and (B) triazoloacridinones. Figure S2: Cytotoxic activity of (A) imidazoacridinones and (B) triazoloacridinones in AR-dependent prostate cancer LNCaP cells. Figure S3: Top 10 GO: Biological process terms (A) and the most representative altered canonical pathways (B) in PCa cells treated with C-1311. Figure S4: Effect of imidazoacridinones and triazoloacridinones on AR transactivation activity measured by luciferase gene reporter assay. Figure S5: Effect of C-1311 on the expression of AR-regulated genes in AR-negative DU-145 cells. Figure S6: Cytotoxic activity of C-1311 and other imidazoacridinones in AR-negative DU-145 cells. Figure S7: Effect of C-1311 on the PCa cell cycle progression. Figure S8: C-1311 induces apoptosis in PCa cells irrespective of AR-status. Table S1: Top 10 deregulated canonical pathways with the list of up- and downregulated genes in LNCaP. Table S2: Top 10 deregulated canonical pathways with the list of up- and downregulated genes in DU-145. Table S3: ‘AR targets upregulated by AR’ gene signature. Table S4: The enriched GO terms in the biological processes related to apoptosis in LNCaP treated with 1 µM for 24 h. Table S5: The enriched GO terms in the biological processes related to apoptosis in DU-145 treated with 1 µM for 24 h.

## Abbreviations

AR	androgen receptor
ARE	androgen response element
ADT	androgen deprivation therapy
BRCA1	breast cancer type 1 susceptibility protein
ChIP	chromatin immunoprecipitation
DDR	DNA damage response
DEG	differentially expressed gene
DHT	dihydrotestosterone
H2AX	histone H2AX
HR	homologous recombination
mCRPC	metastatic castration-resistant prostate cancer
MTT	3-(4,5-diethylthiazol-2-yl)-2,5-diphenyltetrazolium bromide
PARP	poly (ADP-ribose) polymerase
PBS	phosphate buffered saline
PCa	prostate cancer
PSA	prostate-specific antigen
RIN	RNA integrity number
