# Supplementary material for: Anticancer Imidazoacridinone C-1311 is Effective in Androgen-Dependent and Androgen-Independent Prostate Cancer Cells
Source: Biomedicines. 2020 Aug 19;8(9):292. doi: 10.3390/biomedicines8090292 (PMC7555468; doi:10.3390/biomedicines8090292)
Supplement: Supplementary file 1 [file biomedicines-08-00292-s001.pdf]

Anticancer Imidazoacridinone C-1311 is Effective in Androgen-Dependent and Androgen-Independent Prostate Cancer Cells

Magdalena Niemira, Barbara Borowa-Mazgaj, Samuel B. Bader, Adrianna Moszyńska, Marcin Ratajewski, Kaja Karaś, Mirosław Kwaśniewski, Adam Krętowski, Zofia Mazerska, Ester M. Hammond and Anna Skwarska

Supplementary Materials

A

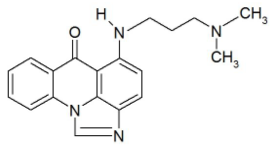
C-1212

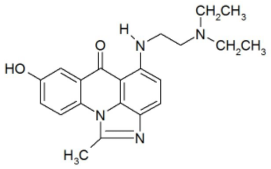
C-1310

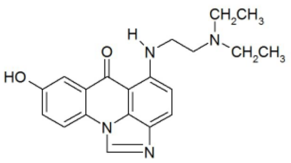
C-1311

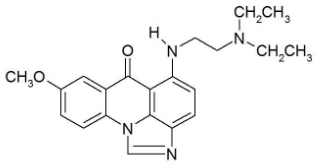
C-1330

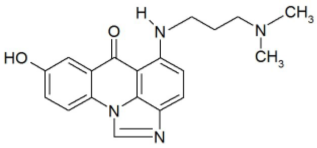
C-1371

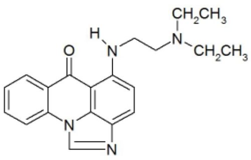
C-1415

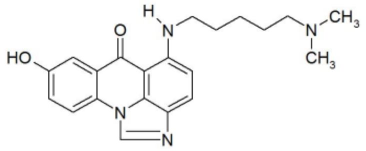
C-1492

B

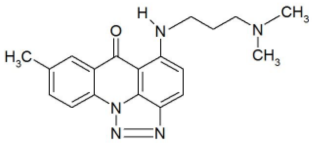
C-1296

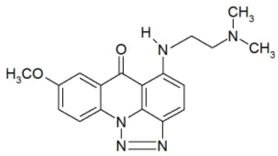
C-1298

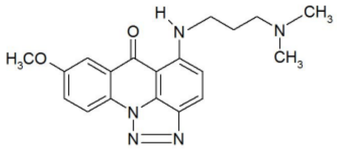
C-1299

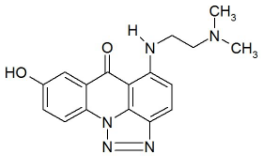
C-1303

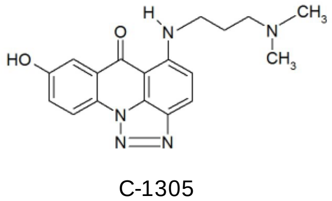
C-1305

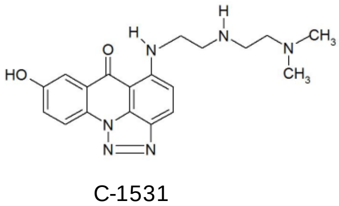
C-1531

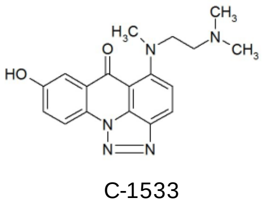
C-1533

Figure S1. Chemical structures of (**A**) imidazoacridinones and (**B**) triazoloacridinones.

A

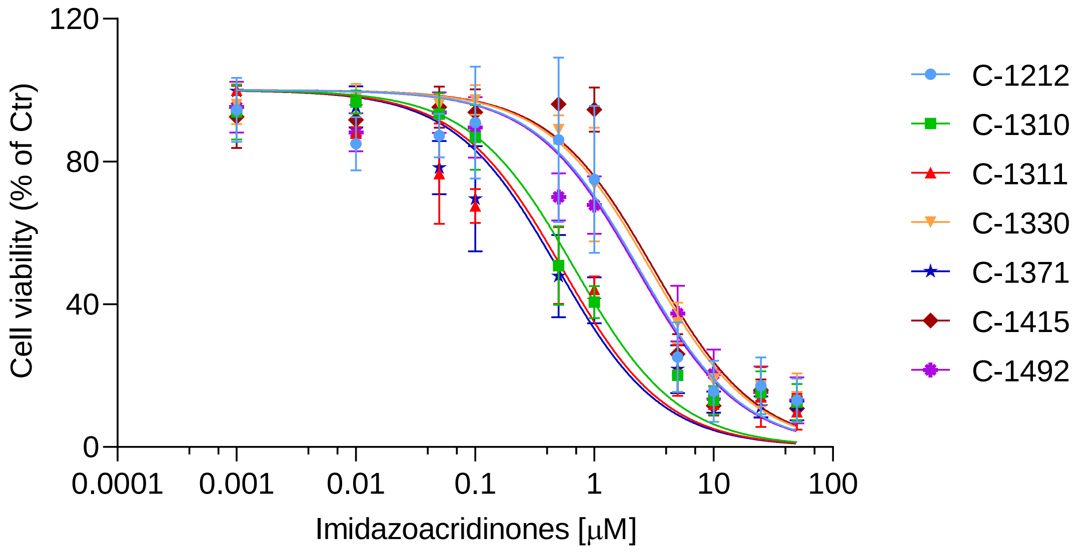

B

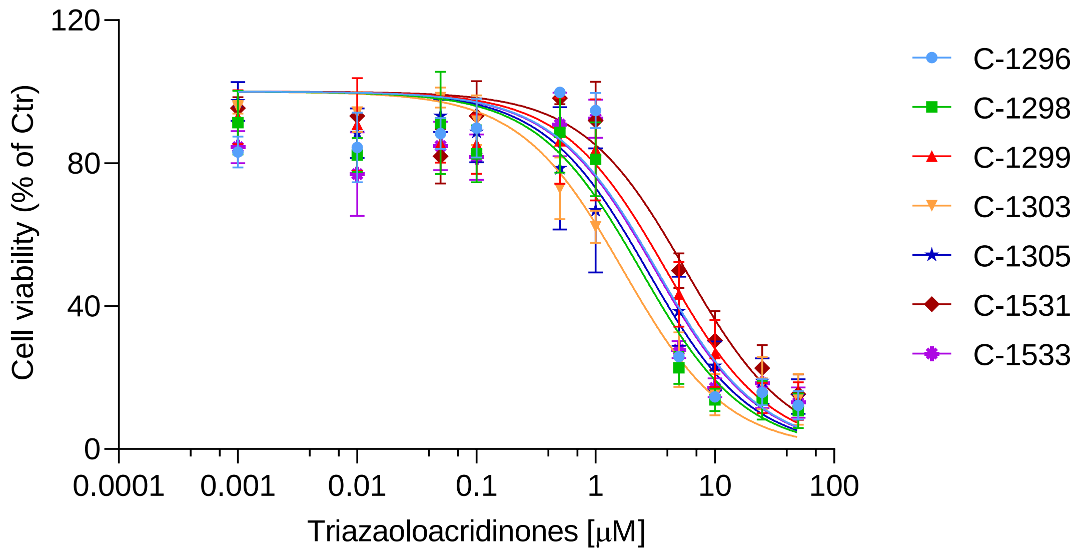

Figure S2. Cytotoxic activity of **(A)** imidazoacridinones and **(B)** triazoloacridinones in AR-dependent prostate cancer LNCaP cells. Cells were treated for 72 h and cell viability was measured using MTT assay. Percent of viable cells was calculated relative to dimethyl sulfoxide (DMSO) treated cells (Ctr). Dose-response curves were prepared using GraphPad Prism 8 software. Data are mean ± SD, $n = 3$.

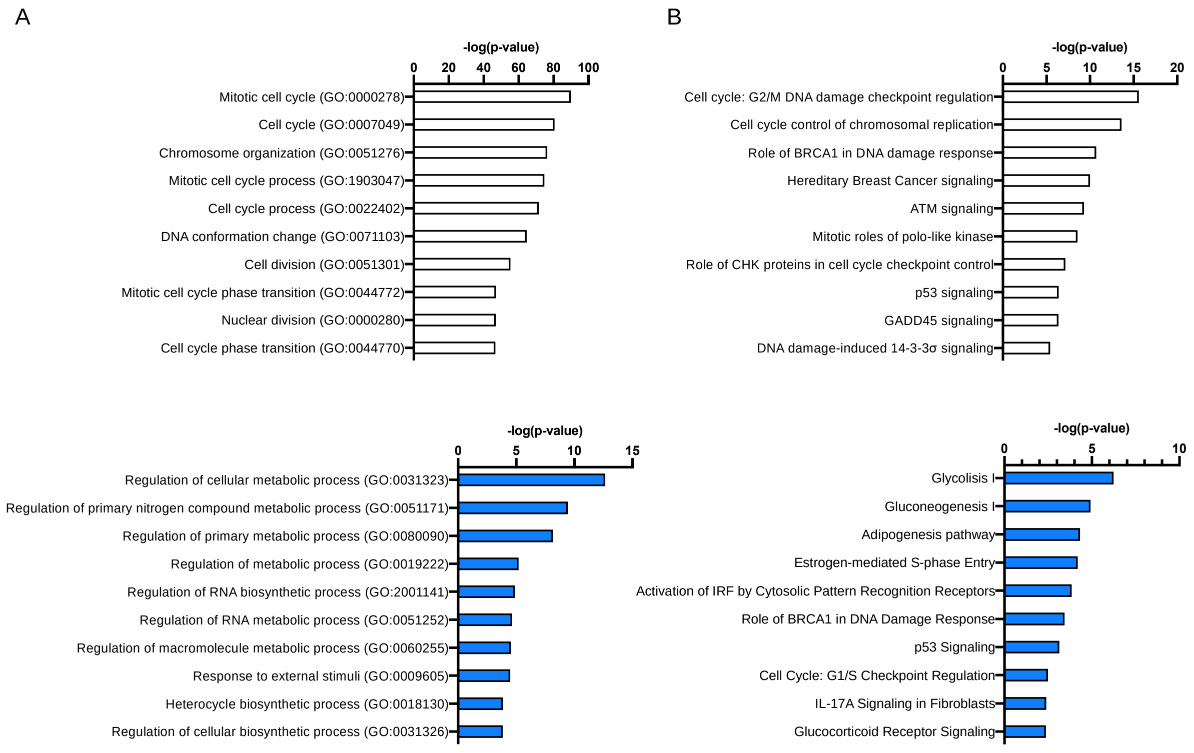

Figure S3. Top 10 GO: Biological process terms **(A)** and the most representative altered canonical pathways **(B)** in PCa cells treated with C-1311. LNCaP and DU-145 cells were treated with 1 μM C-1311 for 24 h and subjected to RNA-seq analysis (n = 3).

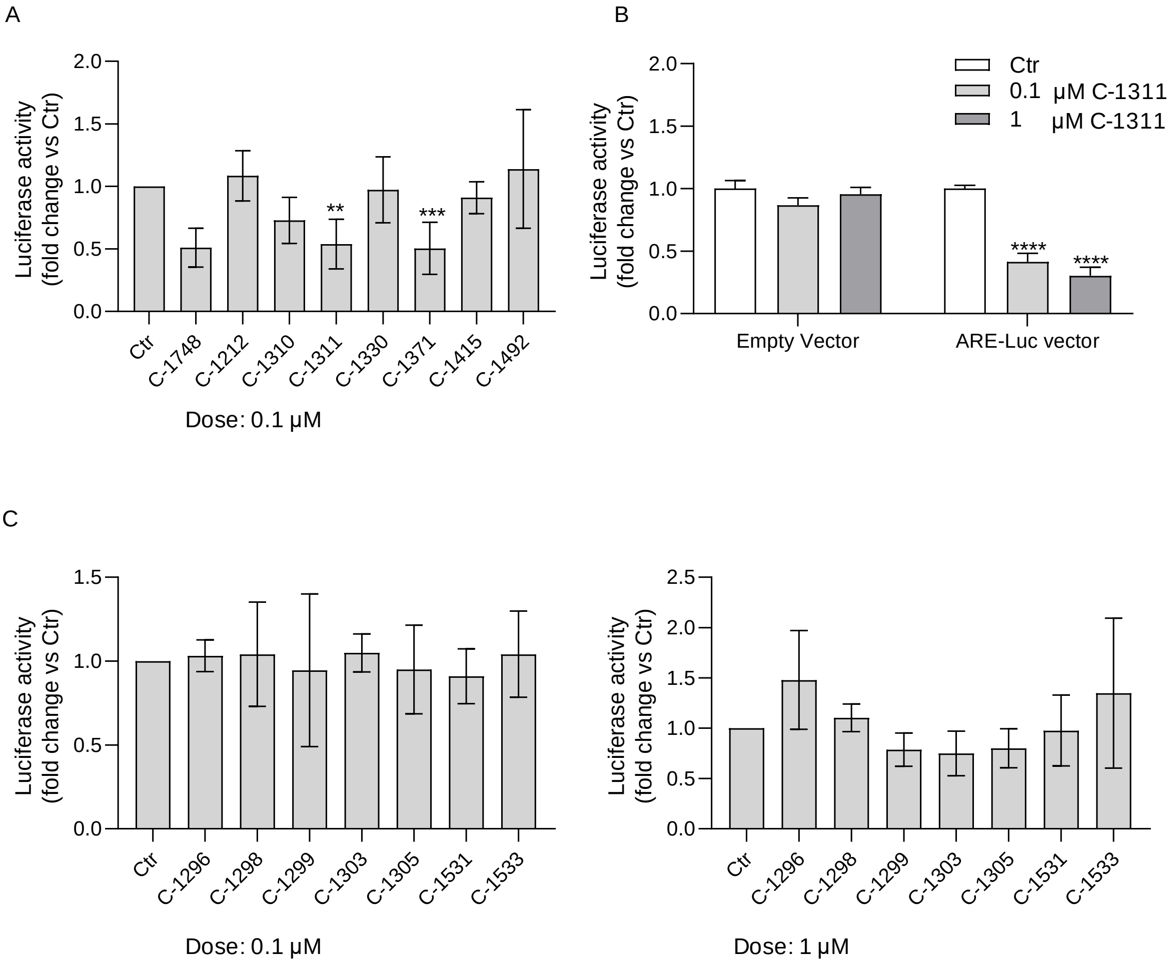

Figure S4. Effect of imidazoacridinones and triazoloacridinones on AR transactivation activity measured by luciferase gene reporter assay. LNCaP-ARE-Luc cells stably expressing luciferase reporter gene linked to AR-responsive promoter were treated with **(A)** imidazoacridinones at 0.1 µM, or **(C)** triazoloacridinones (0.1 or 1 µM) for 24 h. Luciferase activity (fold-change as compared to untreated cells) is shown. Ctr, cells treated with DMSO. Data are mean ± SD, $n = 3$. Significance: one-way Anova test, *** $p < 0.001$, ** $p < 0.01$. **(B)** LNCaP cells were transiently transfected with empty vector or ARE-Luc vector, treated with C-1311 for 24 h and analyzed for luciferase expression. Data are mean ± SD, $n = 3$. Significance: one-way Anova test, **** $p < 0.0001$.

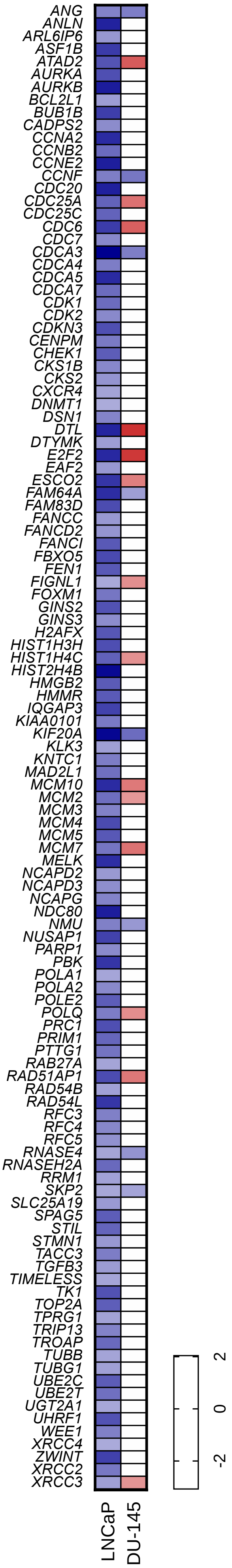
ANG
ANLN
ARL6IP6
ASF1B
ATAD2
AURKA
AURKB
BCL2L1
BUB1B
CADPS2
CCNA2
CCNB2
CCNE2
CCNF
CDC20
CDC25A
CDC25C
CDC6
CDC7
CDCA3
CDCA4
CDCA5
CDCA7
CDK1
CDK2
CDKN3
CENPM
CHEK1
CKS1B
CKS2
CXCR4
DNMT1
DSN1
DTL
DTYMK
E2F2
EAF2
ESCO2
FAM64A
FAM83D
FANCC
FANCD2
FANCI
FBXO5
FEN1
FIGNL1
FOXM1
GINS2
GINS3
H2AFX
HIST1H3H
HIST1H4C
HIST2H4B
HMGB2
HMMR
IQGAP3
KIAA0101
KIF20A
KLK3
KNTC1
MAD2L1
MCM10
MCM2
MCM3
MCM4
MCM5
MCM7
MELK
NCAPD2
NCAPD3
NCAPG
NDC80
NMU
NUSAP1
PARP1
PBK
POLA1
POLA2
POLE2
POLQ
PRC1
PRIM1
PTTG1
RAB27A
RAD51AP1
RAD54B
RAD54L
RFC3
RFC4
RFC5
RNASE4
RNASEH2A
RRM1
SKP2
SLC25A19
SPAG5
STIL
STMN1
TACC3
TGFB3
TIMELESS
TK1
TOP2A
TPRG1
TRIP13
TROAP
TUBB
TUBG1
UBE2C
UBE2T
UGT2A1
UHRF1
WEE1
XRCC4
ZWINT
XRCC2
XRCC3
LNCaP
DU-145
2
0
-2

Figure S5. Effect of C-1311 on the expression of AR-regulated genes in AR-negative DU-145 cells. Cells were treated with DMSO or 1 μM C-1311 for 24 h and subjected to RNA-seq. List of genes in the heat map contains genes identified as DEGs in the 'Androgen receptor targets upregulated by AR' gene signature for LNCaP cells (see Figure 1B). In DU-145 cells, C-1311 treatment did not affect the expression of genes that were significantly repressed in LNCaP cells. Gene expression values for each gene were normalized to the standard normal distribution to generate log fold change (FC). Genes with |log2FC| > 1 and FDR < 0.05 were considered as differentially expressed genes. Data are from three independent experiments.

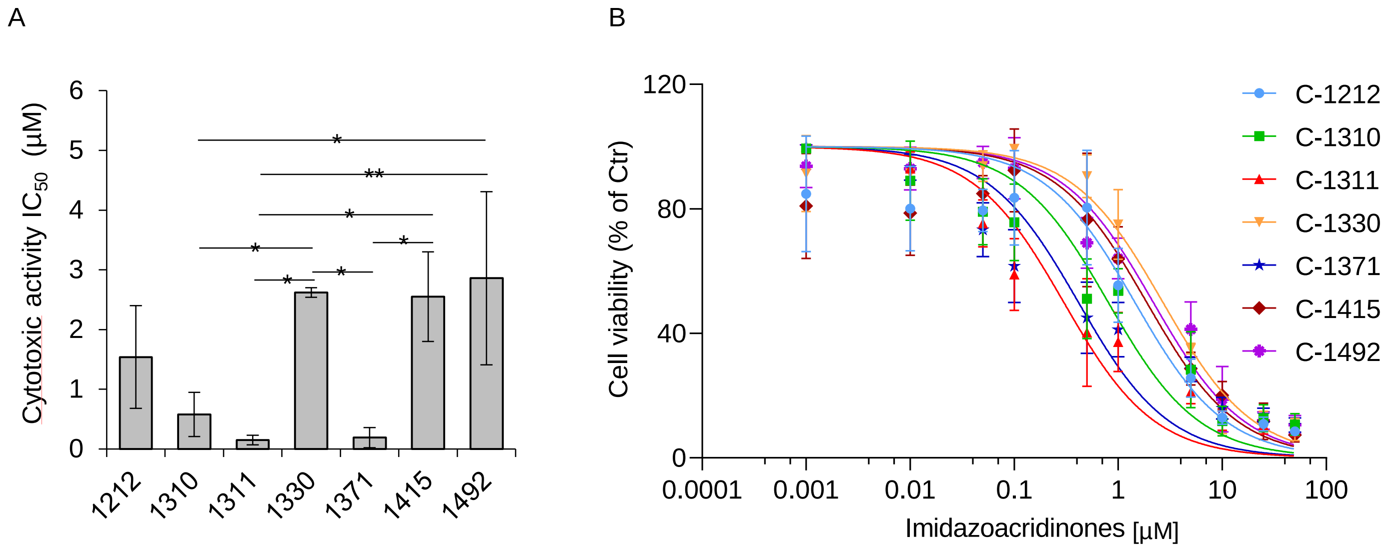

Figure S6. Cytotoxic activity of C-1311 and other imidazoacridinones in AR-negative DU-145 cells. Cells were treated for 72 h and cell viability was measured using MTT assay. Percent of viable cells was calculated relative to dimethyl sulfoxide (DMSO) treated cells (Ctr). **(A)** The IC_{50} values and **(B)** dose-response curves were prepared using GraphPad Prism 8 software. Data are mean ± SD, $n = 3$. Significance: one-way Anova with Tukey's multiple comparison test, ** $p < 0.01$, * $p < 0.05$.

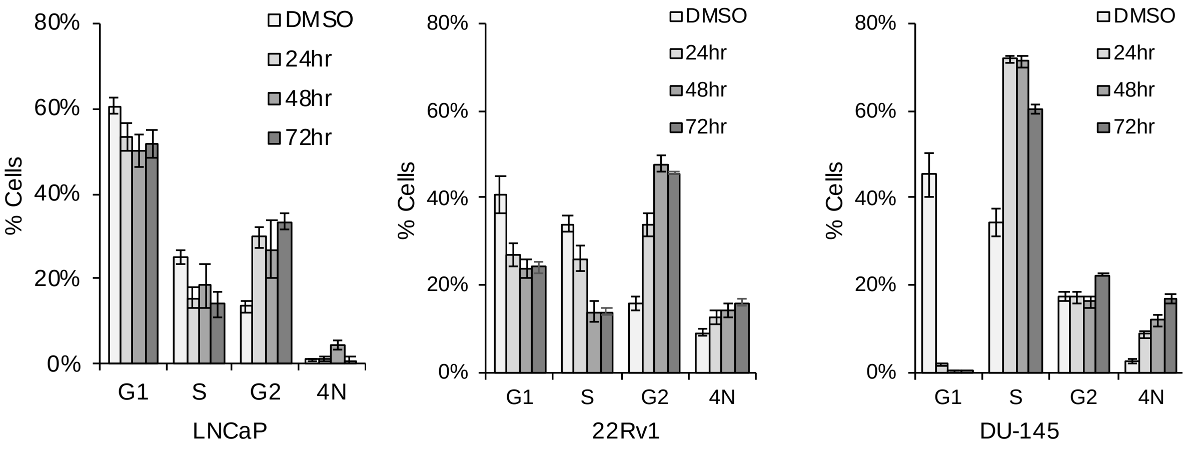

Figure S7. Effect of C-1311 on the PCa cell cycle progression. PCa cells were treated with DMSO or 1 μM C-1311 for 24-72 h, stained with PI and analyzed by flow cytometry. Bar graphs represent the percentage of PCa cells in G1, S, G2/M phase of the cell cycle, and polyploid cells with >4N DNA content. Data are mean ± SD, $n = 3$.

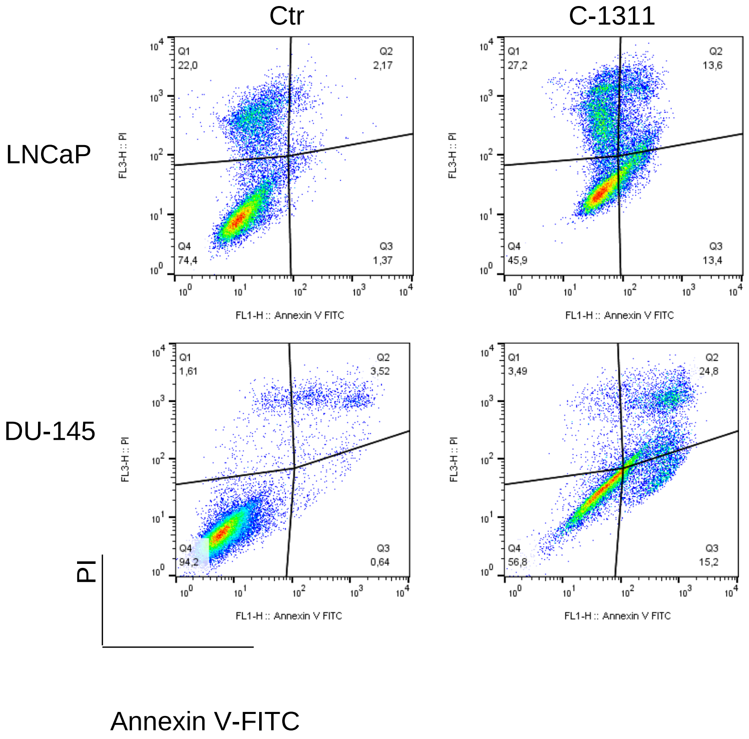

Figure S8. C-1311 induces apoptosis in PCa cells irrespective of AR-status. Cells were treated with DMSO or 1 μM C-1311 for 72 h, stained with Annexin V/PI and analyzed by flow cytometry. Representative plots show live cells (Annexin V-/PI-, Q4), early-apoptotic cells (Annexin V+/PI-, Q3), late-apoptotic cells (Annexin V+/PI+, Q2), and necrotic cells (Annexin V-/PI+, Q1).

Table S1. Top 10 deregulated canonical pathways with the list of up-regulated and down-regulated genes in LNCaP cells.

Ingenuity Canonical Pathways	Up-regulated genes	Down-regulated genes
Cell cycle: G2/M DNA damage checkpoint regulation	*CDKN1A, GADD45A, MDM2*	*BORA, BRCA1, CCNB1, CCNB2, CDK1, CHEK1, CKS1B, PKMYT1, PLK1, TOP2A, WEE1*
Cell cycle control of chromosomal replication	-	*CDC45, CDC6, CDK1, DBF4, DNA2, MCM6, MCM8, ORC1, POLA2, PRIM1, TOP2A*
Role of BRCA1 in DNA damage response	*CDKN1A, GADD45A*	*BLM, BRCA1, CHEK1, E2F1, FAAP24, FANCC, FANCD2, PLK1, RBL1*
Hereditary Breast Cancer Signaling	*CDKN1A, DDB2, GADD45A, XPC*	*BLM, BRCA1, CCNB1, CDK1, CHEK1, E2F1, FAAP24, FANCC, FANCD2, H2AFX, TUBG1, WEE1*
ATM signaling	*CDKN1A, GADD45A, MDM2*	*BLM, BRCA1, CCNB1, CCNB2, CDC25A, CDK1, CHEK1, FANCD2, H2AFX*
Mitotic Roles of Polo-Like Kinase	-	*CCNB1, CCNB2, CDC20, CDC25A, CDK1, PKMYT1, PLK1, PLK4, PTGG1, WEE1*
Role of CHK Proteins in Cell Cycle Checkpoint Control	*CDKN1A*	*BRCA1, CDC25A, CDK1, CDK2, CHEK1, E2F1, PLK1*
Role of CHK Proteins in Cell Cycle Checkpoint Control	*BBC3, CDKN1A, FAS, GADD45A, MDM2*	*BIRC5, BRCA1, CDK2, CHEK1, E2F1, STAG1*
GADD45 signaling	*CDKN1A, GADD45A*	*BRCA1, CCNB1, CDK1, CDK2*
DNA damage-induced 14-3-3σ Signaling	-	*BRCA1, CCNB1, CCNB2, CDK1, CDK2*

Table S2. Top 10 deregulated canonical pathways with the list of up-regulated and down-regulated genes in DU-145 cells.

Ingenuity Canonical Pathways	Up-regulated genes	Down-regulated genes
Glycolysis I	-	*ALDOA, ALDOC, GPI, PGAM1, PGK1, TPI1*
Gluconeogenesis I	-	*ALDOA, ALDOC, GPI, PGAM1, PGK1*
Adipogenesis pathway	*CEBPA, CEPPB, DDIT3, HDAC9, NR1D2, SIRT1, TXNIP, XBP1*	*FGFR4, GTF2H5, HDAC11, SMO*
Estrogen-mediated S-phase Entry	*CDC25A, CDKN1A, MYC, RBL1*	*CCNA1, E2F1*
Activation of IRF by Cytosolic Pattern Recognition Receptors	*CDC25A, CDKN1A, MYC, RBL1*	-
Role of BRCA1 in DNA Damage Response	*BRCA1, CDKN1A, E2F1, GADD45A, MLH1, RBL1, SMARCA4*	-
p53 Signaling	*BBC3, BRCA1, CDKN1A, E2F1, GADD45A, HDAC9, JUN, SIRT1, SNAI2*	*FGFR4*
Cell Cycle: G1/S Checkpoint Regulation	*CDC25A, CDKN1A, E2F1, HDAC9, MYC, RBL1*	*HDAC11*
IL-17A Signaling in Fibroblasts	*CEBPB, JUN, LCN2, NFKBIB, NFKBIE*	-
Glucocorticoid Receptor Signaling	*CDKN1A, CEBPB, FOXO3, HSPA9, IL1RN, JUN, NFKBIB, NFKBIE, SMARCA4, TAF4B, VIPR1*	*FGFR4, GTF2H5, HSPA8, PBX1*

Table S3. 'AR targets upregulated by AR' gene signature.

AADAT, ABCC4, ABCE1, ABHD2, ACSL3, ACTA1, ACTG1, ACTR3, AGR2, AK3L2, AKT1, ALDH1A3, AMD1, ANG, ANLN, ARF4, ARG1, ARG2, ARL6IP6, ARMET, ASF1B, ASPH, ATAD2, AURKA, AURKB, B2M, BCAP29, BCCIP, BCL2L1, BOP1, BRP44, BUB1B, BXDC2, C12orf48, C13orf27, C16orf61, C16orf75, C19orf48, C1orf116, C1orf43, C1orf80, C7orf24, C9orf152, CACYBP, CADPS2, CAMKK2, CCNA2, CCNB2, CCNC, CCNE2, CCNF, CCT6A, CCT7, CDC2, CDC20, CDC25A, CDC25C, CDC6, CDC7, CDCA3, CDCA4, CDCA5, CDCA7, CDH2, CDK1, CDK2, CDK20, CDKN3, CENPM, CENPN, CHEK1, CHORDC1, CHPT1, CKS1B, CKS2, CLEC2D, CMTM7, CNIH, CTNNB1, CXCR4, DBC1, DBI, DC2, DCK, DDX21, DDX39, DEGS1, DHCR24, DHRS3 DKFZP686A01247, DKFZp762E1312, DLG7, DNAJB9, DNAJC9, DNASE2B, DNM1L, DNMT1, DPH2, DSCR2, DSN1, DTL, DTYMK, DUSP4, EAF2, E2F2, EBNA1BP2, EDG7, EEF1B2, EEF1E1, EGFR, EHF, ELL2, ELOVL1, ELOVL5, ENDOD1, EP300, ERBB2, ERBB3, ERGIC2, ERRFI1, ESCO2, ETV1, EVA1, EXOSC3, FADS1, FAM64A, FAM83D, FANCC, FANCD2, FANCI, FASN, FBXO38, FBXO5, FEN1, FGFR3, FIGNL1, FKBP11, FKBP5 , FLJ25416, FOXM1, FUSIP1, FZD6, GAL, GFM1, GHR, GINS2, GINS3, GLRX2, GLYATL2, GSR, GTF3A GUCY1A3, H2AFX, HAT1, HEATR1, HEATR2, HER3, HERPUD1, HIST1H4C, HIST1H3H, HIST2H4B, HK2 HLA-DMA, HM13, HMBS, HMG1L1, HMGB2, HMGCS2, HMMR, HMOX1, HNRPA3, HNRPAB, HOMER2 HPGD, HSP90B1, HSPC111, IDH1, IFI44, IFIT2, IGF1R, IGFBP3, IL8, IMPA2, IMPDH1, IQGAP3, JUN, KCNN2 KDELR2, KIAA0101, KIF20A, KISS1, KISS1R, KLK3, KLK4, KNTC1, KRT18, KRT8, LAMC1, LCP1, LDLR, LIFR, LOC400451, LRGI1, LRP8, LRRFIP2, LYAR, LYPLAL1, MAD2L1, MAGEA4, MALT1, MARS2, MCM10, MCM2, MCM3, MCM4, MCM5, MCM7, MELK, METTL1, MLPH, MPHOSPH9, MPP6, MPZL1, MSTO1, MTP18, MYC NANS, NAT1, NCAPD2, NCAPD3, NCAPG, NDC80, NDRG1, NFKB1A, NKX3-1, NMU, NOL1, NOL5A, NOLC1, NT5DC3, NUSAP1, OBFC2A, ODC1, ORM1, PAK1IP1, PARP1, PASK, PBK, PC, PCNA, PDIA4, PDIA5, PDIA6, PDSS1, PECI, PEX10, PGAM1, PGC, PGM3, PI4K2B, PIAS1, PIGW, PMEPA1, PMM2, POLA1, POLA2 POLE2, POLE3, POLQ, PPAP2A, PPAPDC1B, PPAT, PPFIBP2, PPID, PPIL5, PPRC1, PRC1, PRDX4, PRIM1, PSD3, PSMA1, PSMA6, PSMC4, PTEN, PTRH2, PTTG1, PUS1, RAB27A, RAB4A, RAD51AP1, RAD51C, RAD54B, RAD54L, RANBP1, RAP1GAP, RFC3, RFC4, RFC5, RHOU, RNASE4, RNASEH2A, RPL29, RPL6, RPS15, RPS7 RRM1, RRP15, RRS1, RTN4, S100A11, SASH1, SAT1, SCARB1, SCD, SDF2L1, SEC11C, SEC22C, SEC24D, SEPP1, SERP1, SGK1, SKP2, SLBP, SLC25A15, SLC25A19, SLC33A1, SLC35F2, SLC39A6, SLC43A1, SLC45A3, SMC4L1, SMS, SNAI2, SNX5, SORD, SPAG5, SPDEF, SRP9, SRPRB, SSR1, SSR2, ST6GALNAC1, STEAP4, STIL, STK39 STMN1, STT3A, STT3B, TACC3, TAF9, TCP1, TFB2M, TGFA, TGFB3, TH, THOC3, THOC4, TIMELESS, TIPARP TIPIN, TJP1, TK1, TMED2, TMED7, TMEFF2, TMEM79, TMEPAI, TMPRSS2, TMSL8, TOP2A, TPM3, TPRG1, TRA16, TRIP13, TROAP, TSC2, TSC22D1, TSPAN13, TUBA1A, TUBA3E, TUBA4A, TUBB, TUBG1, TYMS, UAP1, UBE2C, UBE2T, UBIAD1, UCHL3, UCHL5, UCK2, UGDH, UGT2A1, UGT2B10, UGT2B11, UGT2B28, UGT2B7 UHRF1, VAPA, VEGFA, WEE1, WNT7B, XRCC2, XRCC3, XRCC4, ZBTB10, ZBTB16, ZNF239, ZWINT

Table S4. The enriched GO terms in the biological processes related to apoptosis in LNCaP treated with 1 μM for 24 h. Enrichment analysis was performed by the STRING.

PC cells	Term ID	Term descritpion	# DEGs	%	FDR	Up-regulated genes	Down-regulated genes
LNCaP	GO:0072332	Intrinsic apoptotic signaling pathway by p53 class mediator	10	17.2	8.6E-04	*CDIP1, PHLDA3, RPS27L, ZMAT3*	*ATAD5, BRCA2, CDKN1A, CHEK2, E2F1, E2F2,*
	GO:0006915	Apoptotic process	49	5.7	1.9E-03	*ADAMTSL4, BBC3, C12orf5, CDIP1, CDKN1A, FAS, GADD45A, INPP5D, KANK2, LGALS14, PHLD3, PLK3, PTGIS, RPS27L, SNCA, TNFRSF10D, TNFRSF12A, ZMAT3*	*ADORA1, ATAD5, BCL2L12, BIRC5, BRCA1, BRCA2, BUB1, CDCA7, CDK1, CHEK1, CHEK2, CIT, CXCR4, CYP1B1, E2F1, E2F2, EAF2, ESPL1, HMGB2, MCM2, MELK, MTFP1, PARP1, PIM1, PLSCR1, PTH, RELT, TNFRSF1B, TOP2A, TPX2, TRAIP*
	GO:0042981	Regulation of apoptotic process	76	5.1	1.9E-03	*ADAMTSL4, BBC3, BTG2, C12orf5, CDKN1A, CXCR2, DRAXIN, FAS, GADD45A, GDNF, GLS2, INPP5D, KALRN, LGALS14, LGALS3, LHX3, LRP1, MDM2, NEFL, PHLDA3, PLK2, PLK3, PTGIS, RPS27L, RRM2B, SNCA, TNFRSF10D, TP53I3, ZMAT3*	*ACTC1, ADORA1, AGTR2, ANP32E, ATAD5, AURKA, AURKB, BCL2L12, BIRC5, BRCA1, CARD10, CDK1, CDKN2D, CHEK1, CYB1B1, DHRS2, DNMT1, E2F1, ECT2, FAIM3, FIGNL1, HMGB2, IKZF3, KIF14, MAD2L1, MELK, MYB, MYBL2, NRG1, PARP1, PIM1, PLAC8, PLAUR, PLK1, PNMA5, PTH, RELT, SKP2, STIL, TERT, TGFB3, TNFRSF12A, TNFRSF1B, TOP2A, WNT5A, XRCC2*
	GO:0097193	Intrinsic apoptotic signaling pathway	15	10.1	2.0E-03	*BBC3, CDIP1, CDKN1A, PHLDA3, RPS27L, ZMAT3*	*ATAD5, BRCA1, BRCA2, CHEK2, CYB1B1, E2F1, E2F2, MELK, TNFRSF1B*
	GO:0042771	Intrinsic apoptotic signaling pathway in response to DNA damage by p53 class mediator	7	23.3	2.1E-03	*CDIP1, CDKN1A, PHLDA3, RPS27L*	*ATAD5, BRCA2, CHEK2*
	GO:0097190	Apoptotic signaling pathway	22	7.8	2.9E-03	*BBC3, CDIP1, CDKN1A, FAS, PHLDA3, PTGIS, RPS27L, TNFRSF10D, ZMAT3*	*ADORA1, ATAD5, BRCA1, BRCA2, CHEK2, CYB1B1, E2F1, E2F2, MELK, PTH, RELT, TNFRSF12A, TNFRSF1B,*
	GO:0008630	Intrinsic apoptotic signaling pathway in response to DNA damage	10	14.1	3.3E-03	*CDIP1, CDKN1A, PHLDA3, RPS27L*	*ATAD5, BRCA1, BRCA2, CHEK2, E2F1, TNFRSF1B*
	GO:0043066	Negative regulation of apoptotic process	45	5.3	1.2E-02	*BTG2, CDKN1A, CXCR2, DRAXIN, FAS, GDNF, LGALS3, LHX3, LRP1, MDM2, NEFL, PLK2, PLK3, RRM2B, SNCA, TNFRSF10D,*	*ACTC1, ADORA1, ATAD5, AURKA, AURKB, BCL2L12, BIRC5, BRCA1, CDK1, CDKN2D, DHRS2, DNMT1, FAIM3, FIGNL1, HMGB2, KIF14, MAD2L1, NRG1, PIM1, PLAC8, PLAUR, PLK1, PTH, STIL, TERT, TGFB3, TNFRSF1B, WNT5A, XRCC2*

Table S5. The enriched GO terms in the biological processes related to apoptosis in DU-145 treated with 1 μM for 24 h. Enrichment analysis was performed for biological processes by the STRING.

PC cells	Term ID	Term descritpion	# DEGs	%	FDR	Up-regulated genes	Down-regulated genes
DU-145	GO:0097193	Intrinsic apoptotic signaling pathway	26	17.1	8.9E-04	*AEN, ATAD5, BBC3, BRCA1, BRCA2, CDKN1A, CEBPB, DDIT3, E2F1, E2F2, ERN1, HIC1, MLH1, NUPR1, PMAIP1, PPP1R15A, SIRT1, STK11, TNFRSF10B, TRIB3, XBP1, XPA*	*BNIP3, HMOX1, PDK1, TMEM117*
	GO:0097190	Apoptotic signaling pathway	39	13.5	9.5E-04	*AEN, ATAD5, BBC3, BRCA1, BRCA2, CASP8AP2, CCK, CDKN1A, CEBPB, DAPK3, DDIT3, E2F1, E2F2, ERN1, FOXO3, HIC1, IFI27, IFI6, JUN, LY96, MLH1, NGF, NUPR1, PMAIP1, PPP1R15A, SIRT1, STK11, TNFRSF10B, TNFRSF8, TNFRSF9, TRIB3, XBP1, XPA*	*ANXA6, BNIP3, HMOX1, PDK1, PTGIS, TMEM117*
	GO:0042981	Regulation of apoptotic process	128	8.7	2.2E-03	*ACTN2, ALB, ANKLE2, ANKRD1, ANO6, APOH, ARG2, ARHGEF2, ATAD5, ATF3, BBC3, BCL10, BEX2, BIK, BIRC3, BLID, BMP2, BRAF, BRCA1, BTG1, CASP8AP2, CCK, CD274, CDKN1A, CEBPB, CTH, CYLD, DAPK3, DDIT3, DHRS2, DLX1, DRAXIN, DUSP6, E2F1, EDN1, ETS1, FGF21, FIGNL1, FNIP1, FOXO3, GADD45A, GATA6, GCG, GDNF, GRIN2A, HSPA9, IDO1, IFI27, IFI6, IFIT2, IFIT3, IFNB1, IGFBP3, IL12B, IL13, IRF7, JUN, KLF4, LHX3, LTA, MAP2K3, MYC, NGF, NUPR1, PAX8, PDGFRB, PF4, PLK3, PMAIP1, RABGGTB, RHOB, SBK2, SFRP4, SIRT1, SNAI2, SPHK1, ST18, TMC8, TNFAIP3, TNFRSF10B, TNFRSF8, TNFRSF9, TOX3, TRAF1, TSC22D3, TSLP, TXNIP, UBD, UCN, XBP1, XDH, ZC3H8*	*ADM, BNIP3, DHCR24, EGLN3, ELL3, FAIM2, FAM162A, FGD3, GAS6, HMOX1, HPN, HSPA1B, HSPE1, LDHA, LRP1, MAP2K6, MAPT, MGMT, MUC1, NDRG1, NEURL1, NME2, NME4, PLK1, PTGIS, RASSF2, SDF2L1, SFRP1, SGK2, SKP2, SMO, SPDEF, STEAP3, TIAF1, TNFAIP8L1*
	GO:0043065	Positive regulation of apoptotic process	61	10.4	3.8E-03	*ANKRD1, ANO6, ARHGEF2, ATF3, BBC3, BCL10, BIK, BLID, BMP2, BTG1, CASP8AP2, CCK, CD274, CYLD, DAPK3, DDIT3, DUSP6, E2F1, FNIP1, FOXO3, GADD45A, GRIN2A, IDO1, IFI27, IFIT2, IFNB1, IL12B, JUN, LTA, MYC, NGF, NUPR1, PDGFRB, PMAIP1, RHOB, SFRP4, SIRT1, ST18, TNFRSF10B, TNFRSF8, TXNIP, UBD, XBP1, XDH, ZC3H8*	*ADM, BNIP3, EGLN3, FAM162A, FGD3, HMOX1, HSPE1, IGFBP3, LDHA, MAP2K6, MAPT, NEURL1, PTGIS, RASSF2, SFRP1, SPDEF*
	GO:0006915	Apoptotic process	83	9.4	4.8E-03	*AEN, AHR, ATAD5, BBC3, BCL10, BEX2, BIK, BIRC3, BLID, BCL10, BEX2, BIK, BIRC3, BLID, BRCA1, BRCA2, CASP8AP2, CCK, CDKN1A, CEBPB, CSRNP1, DAPK3, DDIT3, E2F1, E2F2, ERN1, FGF21, FOXO3, GADD45A, GLRX2, H1F0, HIC1, IFI27, IFI6, IFIT2, IL24, JUN, LCN2, LTA, LY96, MCM2, MEF2A, MEGF10, MLH1, MX1, MYC, NGF, NUPR1, PLK3, PMAIP1, PPP1R15A, PTPRH, RHOB, SIRT1, STK11, TNFAIP3, TNFRSF10B, TNFRSF19, TNFRSF8, TNFRSF9, TOX3, TRAF1, TRIB3, USP53, XAF1, XBP1, XPA, ZC3H8*	*ANXA6, APLP1, BCL7C, BNIP3, BCL7C, BNIP3, DFFB, DHCR24, EGLN3, FAIM2, FAM162A, GAS6, HK2, HMOX1, IGFBP3, KREMEN1, MAP2K6, PDK1, PTGIS, STEAP3, TIAF1, TMEM117*
	GO:0070059	Intrinsic apoptotic signaling pathway in response to endoplasmic reticulum stress	9	29.0	1.6E-02	*BBC3, CEBPB, DDIT3, ERN1, PPP1R15A, TNFRSF10B, TRIB3, XBP1*	*TMEM117*
	GO:1902041	Regulation of extrinsic apoptotic signaling pathway via death domain receptors	10	19.3	4.8E-02	*ARHGEF2, ATF3, BRCA1, PMAIP1, TMC8, TNFAIP3, TNFRSF10B*	*FAIM2, HMOX1, SFRP1*
